# In situ fenestration of a frozen elephant trunk prosthesis to recanalize the left subclavian artery

**DOI:** 10.1016/j.jvscit.2025.102059

**Published:** 2025-11-21

**Authors:** George Apostolidis, Petroula Nana, Daour Yousef al Sarhan, Lennart Bax, Tilo Kölbel, Giuseppe Panuccio

**Affiliations:** a German Aortic Centre, Department of Vascular Medicine, University Heart and Vascular Centre UKE Hamburg, Hamburg, Germany; b Department of Cardiovascular Surgery, German Aortic Centre Hamburg, University Heart and Vascular Centre Hamburg, Hamburg, Germany

**Keywords:** Electrified wire technique, Endovascular aortic repair, Frozen elephant trunk, In situ fenestration, Left subclavian artery

## Abstract

Maintaining left subclavian artery (LSA) patency is essential for preventing spinal cord ischemia before extensive aortic coverage. We present a case of in situ fenestration on a zone 2 frozen elephant trunk graft to restore the native LSA perfusion after a prior LSA bypass occlusion. Fenestration was performed using the electrified wire technique, followed by balloon-expandable covered stent implantation. The procedure was completed with distal thoracic extensions. The postoperative imaging confirmed LSA patency. This approach offers a feasible, reproducible solution for selected patients requiring LSA recanalization after frozen elephant trunk.

Frozen elephant trunk (FET) represents the indicated approach in fit patients with aortic arch and proximal descending aorta diseases.^1^^,^^2^ The simplified Zone 2 FET has been associated with reduced cerebrovascular morbidity and mortality compared with Zone 3 FET.^3^^,^^4^ During Zone 2 FET, the revascularization of the left subclavian artery (LSA) is performed through a carotid subclavian or bypass between the LSA and aortic graft.^4^ Limited data report an occlusion rate of 6% following Zone 2 FET.^5^ LSA occlusion management may be conservative or interventional, depending on the clinical relevance; LSA revascularization using bypass, transposition, or in situ fenestration is selected most commonly to prevent spinal cord ischemia (SCI) and preserve upper limb perfusion.^1^

Herein, we present an in situ fenestration for LSA recanalization using the electrified wire technique after a zone 2 FET in a patient needing extensive aortic coverage.

## Surgical technique

### Case report

**Fig 1:**
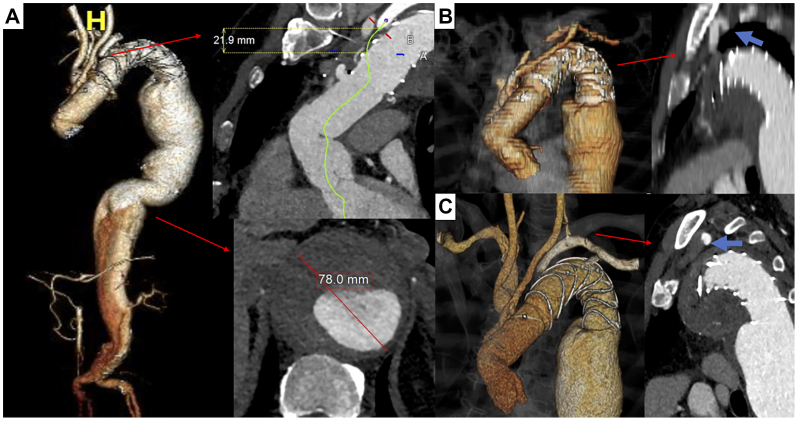
**(A)** A preoperative three-dimensional model demonstrating the extensive type 2 thoracoabdominal aneurysm. The length between ligation and frozen elephant trunk (FET) entry level and the maximal diameter are magnified. **(B)** A three-dimensional aortic model demonstrating the extra-anatomic left subclavian artery (LSA) bypass. Note the compression of the bypass behind the scapula (*blue arrow*). **(C)** A preoperative three-dimensional model demonstrating the FET and the LSA perfused retrogradely from left vertebral artery, due to bypass occlusion (*blue arrow*; lack of contrast enhancement due to occlusion).

A 64-year-old male presented with diffuse abdominal tenderness 3 years after a zone 2 FET (Thoraflex Hybrid; Terumo Aortic) combined with ligation and revascularization of the LSA via a supraclavicular bypass (Fig 1, *A*). An emergent computed tomography angiography revealed a 78-mm type II thoracoabdominal aneurysm (Fig 1, *A*) and a previously unknown occlusion of the LSA bypass (Fig 1, *B* and *C*).

Given his symptoms and multimorbid profile, including heart insufficiency and chronic kidney disease, an urgent endovascular approach, with extensive aortic coverage, was decided, including LSA revascularization for SCI prevention. The native LSA was chosen for recanalization over the existent bypass, due to its lower thrombotic burden and risk of distal embolization and reocclusion. Written informed consent was obtained; anonymized data exempted Ethics Committee approval.

### Technique

Percutaneous access was obtained via both common femoral (CFA) and the left brachial artery. A 10F sheath was inserted into the right CFA and a 4F sheath into the left. A short 6F sheath was inserted into the left brachial artery. Systemic heparinization with a targeted activation clotting time >250 seconds and near-infrared spectroscopy were applied.

**Fig 2:**
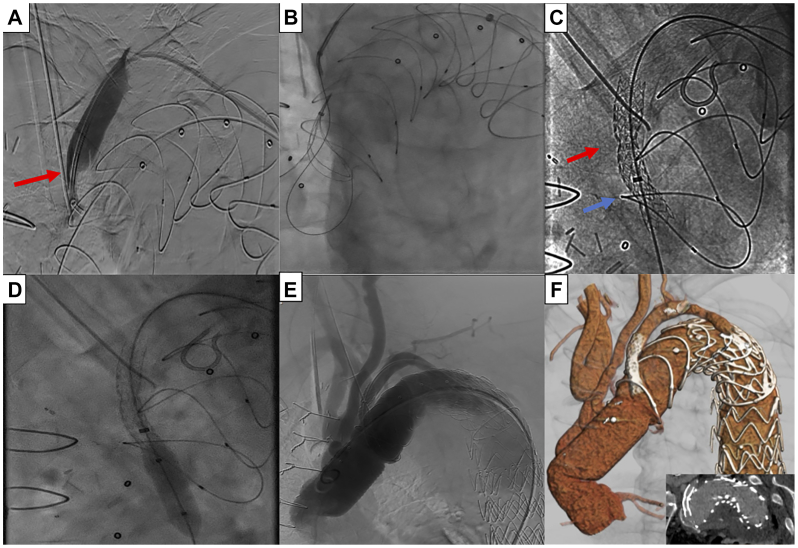
**(A)** Angiography confirming the overcome of the left subclavian artery (LSA) ligation point (*red arrow*). **(B)** Snaring of the advanced wire through the newly introduced fenestration. **(C)** Image after the deployment of the second balloon expandable covered stent (*red arrow*, distal stenotic area-ligation site; *blue arrow*, proximal stenotic area in situ fenestration). **(D)** Balloon dilation from left brachial artery access to achieve antegrade orientation of the bridging stents. **(E)** Final angiography of the proximal aorta revealing patency and lack of target vessel-related endoleak for LSA. **(F)** Postoperative three-dimensional image of the aorta revealing patency and lack of target vessel-related endoleak for LSA.

From the right CFA, a tulip snare (EnSnare; Merit Medical) was advanced into the aortic arch. A 6F × 55 cm sheath and a 5F Berenstein catheter (Tempo; Cordis) were advanced from the left brachial access above the LSA ligation point. A 0.014′ Astato XS20 wire (Asahi Intecc, Seto-shi) was passed across the ligation point, followed by the catheter and sheath (Fig 2, *A*). The Astato wire’s polytetrafluoroethylene (PTFE) was stripped distally and connected to the electrocautery (70 watts; cutting mode).

After ensuring the perpendicular sheath position on the FET dome, an in situ fenestration was performed using the electrified wire.^6^ After penetrating the graft, the wire was snared from the right CFA to establish a through-and-through access (Fig 2,*B*). Using the brachial access, fenestration balloon dilation was performed (5 × 20 mm; Viatrac 14 Plus Peripheral Dilation Catheter; Abbott). A 7F × 90 cm sheath (Check-Flo Performer, Cook Medical LLC) was advanced from the right CFA, followed by a balloon-expandable covered stent (Advanta V12; 6 × 38 mm; Getinge/Atrium Medical Corporation), deployed and flared at 8 mm.

Two stenoses of the deployed covered stent related to a potential recoil of the Dacron graft and prior LSA ligation were remarked. A concomitant insufficient bridging stent protrusion into the FET graft led to the deployment of an additional Advanta V12 6 × 38 mm, postdilated to 8 mm with a high-pressure balloon, and flared to 10 mm using the upper access, to achieve an anterograde bridging stent orientation (Fig 2, *C* and *D*). A selective angiography confirmed the LSA’s perfusion and no endoleak (Fig 2, *E*). The procedure was completed after the implantation of three thoracic and a physician-modified endograft targeting an intercostal artery.

### Postoperative course

The patient was extubated and transferred to the intensive care unit. No sign of SCI was detected. His further course was complicated by bowel ischemia (chronic superior mesenteric artery occlusion, unrelated to the index procedure) and acute pancreatitis, hampering the completion of the repair. A 6-week computed tomography angiography confirmed the LSA patency (Fig 2, *F*). Further follow-up and completion of the repair would be performed after the improvement of patient’s clinical status.

## Discussion

Despite proximal FET, up to 60% of patients may experience distal aortic disease progression, requiring further coverage.^3^ In these cases, LSA patency is crucial for SCI prevention.^7^ Although Zone 2 FET is considered an improved alternative, no LSA revascularization approach has been established as optimal.^2^^,^^4^ In the present case, a bypass connecting the third branch of the FET to the LSA transition point to the axillary artery via a supraclavicular exposure of the LSA was chosen but occluded later on.^4^ In such cases, LSA revascularization may be achieved by recanalizing the existent bypass, establishing a new bypass or transposition, or recanalizing the native LSA through in situ fenestration. Thrombus burden, anatomical details, patient’s clinical status, and surgeon’s preference may affect the final decision.^5^^,^^8^ In situ fenestration was selected in this case to minimize the risk of distal thrombus embolization and reocclusion due to its unfavorable position, being compressed between the aorta and scapula (Fig 1, *B*).

In situ fenestration has mainly been utilized during urgent endovascular procedures for aortic side branches preservation.^9^^,^^10^ A retrograde fenestration via the brachial access was preferred, as a fenestration creation from the inner surface of the FET could be risky, due to misidentification of the occluded LSA orifice and challenging perpendicular orientation of the steerable sheath to the dome of the graft.^11^ Double perpendicular projections were used to ensure the accurate Astato wire orientation and avoid fabric tears.^11^ Applying the electrified wire distal to the first metal ring of the endovascular portion of the FET, along with dextrose infusion during this process; to limit electrothermal energy dispersion, ensured avoiding any thermal injury of the distal anastomotic graft segment.^6^

The woven Dacron configuration provides durability but may compromise fenestration patency due to recoil.^10^ After deployment of the first bridging stent, two stenoses at the levels of prior ligation and Dacron perforation necessitated an additional covered stent implantation and high-pressure balloon postdilation to ensure patency.^10^ Postdilation and/or reinforcement may be needed in cases of intraoperatively detected recoil during in situ fenestration.^10^ Although the long-term patency remained unassessed, the short-term patency ensured SCI prevention. Regarding in situ fenestration of PTFE endografts, although clear evidence for the electrified wire technique is lacking, previous reports have shown variable outcomes; with PTFE being more vulnerable to larger fabric tears and fenestration deformation through time.^12^

The “electrified wire” technique has been successfully applied in various endovascular procedures and offers a safe, cost-effective method for in situ fenestration, particularly compared with laser-based approaches.^6^^,^^8^ The low-profile 0.014′ Astato permitted crossing the highly stenotic LSA ligation point, whereas its stainless steel core provided enough support to cross the thrombus/debris between the FET graft and aortic wall.^6^ To account for potential dissection or rupture during catheterization and fenestration creation maneuvers, an appropriately sized thoracic endograft and a balloon-expandable covered stent were available for further endovascular management. If the in situ fenestration failed, the existent bypass recanalization, despite the embolization risk, or alternatively, a carotid subclavian bypass could have been pursued.

## Conclusions

In situ fenestration using an electrified wire may represent a feasible and reproducible approach for LSA revascularization after zone 2 FET, employing readily available materials of the routine clinical practice.

## Funding

None.

## Disclosures

T.K. is a consultant and proctor for and has intellectual property with Cook Medical, receiving royalties, speaking fees, and research, travel, and educational grants. The remaining authors report no conflicts.

## References

[1] Czerny M., Schmidli J., Adler S. (2019). Current options and recommendations for the treatment of thoracic aortic pathologies involving the aortic arch: an expert consensus document of the European Association for Cardio-Thoracic surgery (EACTS) and the European Society for Vascular Surgery (ESVS). Eur J Cardiothorac Surg.

[2] Papakonstantinou N.A., Martinez-Lopez D., Chung J.C.Y. (2024). The frozen elephant trunk: seeking a more definitive treatment for acute type A aortic dissection. Eur J Cardiothorac Surg.

[3] Detter C., Bax L., Panuccio G. (2025). Complicated acute type A aortic dissection and severe aortic atherosclerosis predict early mortality after frozen elephant trunk procedure. Eur J Cardiothorac Surg.

[4] Detter C., Demal T.J., Bax L. (2019). Simplified frozen elephant trunk technique for combined open and endovascular treatment of extensive aortic diseases. Eur J Cardiothorac Surg.

[5] Mattern L., Pfeiffer P., Wittemann K. (2025). Extra-anatomic left subclavian artery bypass patency in frozen elephant trunk surgery. JTCVS Tech.

[6] Torrealba J.I., Panuccio G., Rohlffs F. (Apr 10, 2024). The electrified wire technique in complex aortic interventions: a case series. J Endovasc Ther..

[7] Chen X., Wang J., Premaratne S., Zhao J., Zhang W.W. (2019). Meta-analysis of the outcomes of revascularization after intentional coverage of the left subclavian artery for thoracic endovascular aortic repair. J Vasc Surg.

[8] Shehab M., Wanhainen A., Tegler G., Mani K., Kuzniar M. (2024). In situ laser fenestration of the thoraflex hybrid frozen elephant trunk for emergent revascularization of the left subclavian artery and laser fenestration for spinal cord perfusion. J Vasc Surg Cases Innov Tech.

[9] Houérou T.L., Nana P., Pernot M. (2023). Systematic review on in situ laser fenestrated repair for the endovascular management of aortic arch pathologies. J Clin Med.

[10] Grima M.J., Wanhainen A., Lindström D. (2024). In situ laser fenestration technique: bench-testing of aortic endograft to guide clinical practice. J Endovasc Ther.

[11] Riga C.V., Bicknell C.D., Basra M., Hamady M., Cheshire N.J.W. (2013). In vitro fenestration of aortic Stent-Grafts: implications of puncture methods for in situ fenestration durability. J Endovasc Ther.

[12] He Y., Wang Y., Zhou X., Wu Z., Zhang H., Li D. (2024). Effects of long term fatigue cycling on in situ fenestrations of polyethylene terephthalate and expanded polytetrafluorethylene thoracic aortic stent grafts: an experimental Study. Eur J Vasc Endovasc Surg.

